# Supplementary material for: Availability and use of personal protective equipment and satisfaction of healthcare professionals during COVID-19 pandemic in Addis Ababa, Ethiopia
Source: Arch Public Health. 2021 Aug 17;79:146. doi: 10.1186/s13690-021-00668-3 (PMC8369137; doi:10.1186/s13690-021-00668-3)
Supplement: Supplementary file 1 — Additional file 1:. Questionnaires for assessment of the availability and utilization of PPE healthcare professionals in public hospitals in Addis Ababa, June 2020. [file 13690_2021_668_MOESM1_ESM.pdf]

Availability and use of personal protective equipment and satisfaction of healthcare professionals during COVID-19 pandemic in Addis Ababa, Ethiopia

Wakgari Deressa[1*], Alemayehu Worku[1], Workeabeba Abebe[2], Muluken Gizaw[1], Wondwossen Amogne[3]

[1]Department of Preventive Medicine, School of Public Health, College of Health Sciences, Addis Ababa University, Addis Ababa, Ethiopia

[2]Department of Pediatrics and Child Health, School of Medicine, College of Health Sciences, Addis Ababa University, Addis Ababa, Ethiopia

[3]Department of Internal Medicine, School of Medicine, College of Health Sciences, Addis Ababa University, Addis Ababa, Ethiopia

***Corresponding author**
Email: deressaw@gmail.com

Questionnaire for Assessment of Availability and Use of Personal Protective Equipment by Healthcare Workers at Public Hospitals in Addis Ababa

Self-Administered Questionnaire

1. Name of the Hospital: ____
2. What is your gender? 1. Male 2. Female
3. What is your age (years)? _______
4. How long (years of experience) have you served in this hospital? ________
5. What is your medical profession?
   1. General Practitioner
   2. Specialist
   3. Resident
   4. Intern
   5. Nurse
   6. Midwife
   7. Health officer
   8. Radiographer
   9. Lab technologist
   10. Other (specify) ________________________________
6. In which Department/unit do you currently work in this hospital?
   1. Medical
   2. Pediatrics
   3. Surgical
   4. Gyn & Obs
   5. OPD
   6. Intensive care
   7. Emergency
   8. Screening/Triage
   9. Isolation room/ward
   10. Anesthesia
   11. Other (please specify) ____
7. What forms of the following PPE were FREQUENTLY **AVAILABLE** in the routine care of patients in your hospital **before** the COVID-19 pandemic? (*Circle all that apply*)

7.1 Gloves	1. Yes 2. No
7.2 Gowns	1. Yes 2. No
7.3 Facemask	1. Yes 2. No
7.4 N95 respirator	1. Yes 2. No
7.5 Eye protection (Goggles and face shield)	1. Yes 2. No
7.6 Hair covers	1. Yes 2. No

   7.7 Other (please describe) _______________________________________

8. What forms of the following PPE did you FREQUENTLY **USE** in your hospital **before** the COVID-19 pandemic? (*Circle all that apply*)

8.1 Gloves	1. Yes 2. No
8.2 Gowns	1. Yes 2. No
8.3 Facemask	1. Yes 2. No
8.4 N95 respirator	1. Yes 2. No
8.5 Eye protection (Goggles and face shield)	1. Yes 2. No
8.6 Hair covers	1.Yes 2. No

   8.7 Other (please describe) _______________________________________

9. What forms of the following PPE were FREQUENTLY **AVAILABLE** to use in your hospital **during** the COVID-19 pandemic? (*Circle all that apply*)

9.1 Gloves	1. Yes 2. No
9.2 Gowns	1. Yes 2. No
9.3 Facemask	1. Yes 2. No
9.4 N95 respirator	1. Yes 2. No
9.5 Eye protection (Goggles and face shield)	1. Yes 2. No
9.6 Hair covers	1. Yes 2. No

9.7 Other (please describe) ______________________________________

10. What forms of the following PPE did you FREQUENTLY **USE** in your hospital **during** the COVID-19 pandemic? (*Circle all that apply*)

10.1 Gloves	1. Yes 2. No
10.2 Gowns	1. Yes 2. No
10.3 Facemask	1. Yes 2. No
10.4. N95 respirator	1. Yes 2. No
10.5 Eye protection (Goggles and face shield)	1. Yes 2. No
10.6 Hair covers	1. Yes 2. No

10.7 Other (please describe) ______________________________________

11. What forms of the following PPE did you **USE** during your last interaction with a patient in your hospital? (*Circle all that apply*)

11.1 Gloves	1. Yes 2. No
11.2 Gowns	1. Yes 2. No
11.3 Facemask	1. Yes 2. No
11.4. N95 respirator	1. Yes 2. No
11.5 Eye protection (Goggles and face shield)	1. Yes 2. No
11.6 Hair covers	1. Yes 2. No

11.7 Other (please describe) ______________________________________

12. Are you satisfied with the current availability of PPE in your hospital during the COVID-19 pandemic?
    1. Very satisfied
    2. Somewhat satisfied
    3. Average
    4. Somewhat unsatisfied
    5. Unsatisfied

13. Are you satisfied with the current use of PPE by health professionals in your hospital?
    1. Very satisfied
    2. Somewhat satisfied
    3. Average
    4. Somewhat unsatisfied
    5. Unsatisfied

14. Are some of the PPE, you would normally use currently, unavailable in your hospital?
    1. Yes 2. No 3. Don't know [***If "No" or "Don't know", please skip to 16***]

15. If YES question 14, please mention them ____

16. Are some of the PPE, you don't normally use, currently available in your hospital?
    1. Yes 2. No 3. Don't know [***If "No" or "Don't know", please skip to 18***]

17. If YES to question 16, please mention them ____

18. Have you received any training in PPE during the past two years? 1. Yes 2. No

19. Have you received training in PPE since the COVID-19 pandemic? 1. Yes 2. No
[*If "No", please skip to 21*]

20. If YES to question 19, what training have you received with regard to PPE since the COVID-19 pandemic? (*Circle all that apply*)

20.1 Gloves	1. Yes 2. No
20.2 Gowns	1. Yes 2. No
20.3 Facemask	1. Yes 2. No
20.4 N95 respirator	1. Yes 2. No
20.5 Eye protection (Goggles and face shield)	1. Yes 2. No
20.6 Hair covers	1. Yes 2.No
20.7 Other (please describe) ____	

21. Have you received training in the cleaning and disinfection procedures of PPE during the current COVID-19 pandemic? 1. Yes 2. No

22. The correct PPE (as recommended by WHO) is always available to me when treating a non-COVID-19 patient in my hospital?
    1. Strongly agree
    2. Agree
    3. Neither agrees nor disagrees
    4. Disagree
    5. Strongly disagree

23. The correct PPE (as recommended by WHO) is always available to me when treating a patient with suspected or confirmed COVID-19 in my hospital?
    1. Strongly agree
    2. Agree
    3. Neither agrees nor disagrees
    4. Disagree
    5. Strongly disagree

24. Do you feel that the PPE currently available to you by your hospital is adequate to protect you when managing patients with suspected or confirmed COVID-19?
    1. Yes 2. No

25. Are you using any "homemade", "creative" PPE such as homemade fabric, cloth face covering, sewed cotton mask, plastic or surgical drapes in a hospital?
    1. Yes 2. No

26. How worried are you about the current availability of PPE in your hospital?
    1. Extremely worried
    2. Generally worried
    3. Neither worried nor not worried
    4. Generally not worried
    5. Not worried at all

27. Have you ever previously provided direct clinical care to any patients affected by an infectious disease outbreak? (Examples of outbreaks: ebola, SARS, MERS, swine flu, avian flu, cholera, zika virus) 1. Yes 2. No

28. Have you ever provided direct clinical care to any suspected or confirmed COVID-19 patient since the 1st report of the case in Ethiopia?
    1. Yes 2. No [*If "No", please skip to 35*]

29. If YES to question 33, how many suspected or confirmed cases of COVID-19 have you had direct clinical contact with since the 1st report of the case in Ethiopia? _______________

30. How do you feel on your preparedness to provide direct care to suspected or confirmed cases of COVID-19?
    1. Completely unprepared
    2. Somewhat unprepared
    3. Neither unprepared or prepared
    4. Somewhat prepared
    5. Very prepared

Thank you so much for your cooperation and time!
