# Supplementary material for: Availability and use of personal protective equipment and satisfaction of healthcare professionals during COVID-19 pandemic in Addis Ababa, Ethiopia
Source: Arch Public Health. 2021 Aug 17;79:146. doi: 10.1186/s13690-021-00668-3 (PMC8369137; doi:10.1186/s13690-021-00668-3)
Supplement: Supplementary file 2 — Additional file 2:. Guidelines for data collectors and supervisors of self-administered questionnaires at public hospitals in Addis Ababa, June 2020. [file 13690_2021_668_MOESM2_ESM.pdf]

Availability and use of personal protective equipment and satisfaction of healthcare professionals during COVID-19 pandemic in Addis Ababa, Ethiopia

Wakgari Deressa[1*], Alemayehu Worku[1], Workeabeba Abebe[2], Muluken Gizaw[1], Wondwossen Amogne[3]

[1]Department of Preventive Medicine, School of Public Health, College of Health Sciences, Addis Ababa University, Addis Ababa, Ethiopia

[2]Department of Pediatrics and Child Health, School of Medicine, College of Health Sciences, Addis Ababa University, Addis Ababa, Ethiopia

[3]Department of Internal Medicine, School of Medicine, College of Health Sciences, Addis Ababa University, Addis Ababa, Ethiopia

***Corresponding author**
Email: deressaw@gmail.com

Guidelines for Data Collectors and Supervisors of Self-Administered Questionnaire at Public Hospitals in Addis Ababa, Ethiopia

June 2020

1. Background

The ongoing COVID-19 pandemic has caused over 5.6 million confirmed cases and claimed over 355,000 lives worldwide as of 26th May 2020. In Ethiopia, the pandemic that started on 13th March 2020 has caused 1,344 confirmed COVID-19 cases with 14 fatal cases by 2nd June 2020. There were 231 recoveries and 1,097 active cases. Despite efforts to contain and mitigate the transmission of the virus, the coronavirus has continued to spread to different parts of Ethiopia at an alarming rate and new more cases have continued to emerge. The people most at risk of infection are those who are in close contact with a COVID-19 patient or who care for COVID-19 patients.

Healthcare workers play a critical role, not only in the clinical management of COVID-19 patients, but also in ensuring adequate infection prevention and control measures in healthcare settings. They are at significant risk of acquiring the infection than the general population; therefore, they are required to protect themselves and prevent transmission in the healthcare settings. One way of preventing infection among healthcare workers is to use Personal Protective Equipment (PPE), such as protective clothing, gloves, masks, and goggles to prevent contamination of the worker. The use of PPE by healthcare workers during the current COVID-19 pandemic is highly recommended based on the national and international safety protocols for healthcare workers. However, basic protective equipment and safety protocols are not always available in many healthcare facilities dealing with COVID-19 patients particularly in low income countries such as Ethiopia. Many healthcare professionals in many healthcare settings have limited access to effective safety measurements to protect their health in the workplace. In addition, compliance with guidance on the correct use of PPE in healthcare setting is another challenge. The current situation of COVID-19 calls for an urgent research that addresses the needs of healthcare workers with regard to their perceptions about the pandemic and the use of PPE. There is generally a lack of sufficient PPE for healthcare workers and healthcare facilities. The purpose of this study is to assess and evaluate the perceptions and practices about personal safety measures among healthcare workers practicing in hospitals in Addis Ababa during the current COVID-19 pandemic.

2. Purpose of the guideline

This guideline is intended to guide data collectors and supervisors for data collection, quality assurance of data and ethical conduct during implementation of the study on PPE by healthcare workers practicing in seven hospitals in Addis Ababa.

3. Study hospitals and sample size allocations

This study will be carried out in seven public hospitals in Addis Ababa city administration in May-June 2020. This includes (1) Tikur Anbessa Specialized Hospital (TASH), (2) Zewditu Memorial Hospital (ZMH), (3) Ghandi Memorial Hospital (GMH), (4) Menelik II Hospital, (5) Yekatit 12 Hospital Medical College, (6) St. Paul Hospital Millennium Medical College, and (7) St. Peter Hospital. The latter two hospitals have currently finalized all the necessary preparations

for admitting and treating COVID-19 patients in addition to Ekka Kotebe General Hospital. Healthcare workers working at these hospitals will constitute the study population. In this study, healthcare worker is defined as all health professionals in the selected hospitals involved in the provision of healthcare services. This includes intern doctors, residents, general practitioners, specialists and sub-specialists, health officers, anesthetists, nurses, midwifes, laboratory technologists, radiologists, physiotherapists, x-ray and laboratory technicians. Although not the target of this study, we are aware that allied health workers, and auxiliary health workers (e.g. cleaning and laundry personnel, clerks, social workers, cleaners, and patient transporters) in the hospitals are also most at risk of infection.

The total sample size required for this survey is 1,500 healthcare workers, divided into the seven hospitals proportional to the size of the healthcare workers practicing in the hospital at the time of the survey. Within the selected hospital, the allocated sample size will also be proportionally divided into the different department/units of the hospital based on the number of healthcare workers working in each department/unit. This study targets healthcare workers at OPD, general ward, emergency ward, medical ward, surgical ward, Gyn&Obs ward, pediatrics ward, intensive care, Screening/Triage/Isolation room, anesthesia, emergency and other wards. Table 1 shows the lists of the hospitals and the sample size allocated to each of them.

Table 1. List of hospitals, sample sizes allocated and data collectors

No.	***Name of Hospital***	***Wards***	***# healthcare workers***	***Sample size***	***Data Collector (DC)***
1	TASH	OPD			Name: Yonas Abebe<br>Phone: 0944162181<br>Code: DCH1
		General ward			
		Emergency ward			
		Medical ward			
		Surgical ward			
		Gyn&Obs ward			
		Pediatrics			
		Other			
		Total		300	
2	Zewditu	OPD			Name: Shibabaw Yirsaw<br>Phone: 0925334474<br>Code: DCH2
		General ward			
		Emergency ward			
		Medical ward			
		Surgical ward			
		Gyn&Obs ward			
		Pediatrics			
		Other			
		Total		200	
3	Ghandi				Name: Sirak T/Mariam<br>Phone: 0911473920<br>Code: DCH3
		Total		150	
4	Yekatit 12	OPD			Name: Yeabsira Mesfin<br>Phone: 0945618645<br>Code: DCH4
		General ward			
		Emergency ward			
		Medical ward			
		Surgical ward			
		Gyn&Obs ward			
		Pediatrics			
		Other			
		Total		200	
5	Menelik II	OPD			Name: Rita Petros<br>Phone: 0916283488<br>Code: DCH5
		General ward			
		Emergency ward			
		Medical ward			
		Surgical ward			
		Gyn&Obs ward			
		Pediatrics			
		Other		200	
		Total			
6	St. Paul	OPD			Name: Fentayehu Abebil<br>Phone: 0953915319<br>Code: DCH6
		General ward			
		Emergency ward			
		Medical ward			
		Surgical ward			
		Gyn&Obs ward			
		Pediatrics			
		Other			
		Total		250	
7	St. Peter	OPD			Name: G/Tsadik Kaleb<br>Phone: 0918604336<br>Code: DCH6
		General ward			
		Emergency ward			
		Medical ward			
		Surgical ward			
		Gyn&Obs ward			
		Pediatrics			
		Other			
		Total		200	
	Total			1,500	07

4. Data collection procedures

The data for this survey will be collected using a paper-based self-administered questionnaire. A self-administered questionnaire (SAQ) refers to a questionnaire that has been designed specifically to be completed by a respondent without intervention by a trained data collector or interviewer collecting the data. That means there is no need of interviewer to motivate or help the respondent to complete the questionnaire. Traditionally the SAQ has been distributed in person or by mail to large groups, but now SAQs are being used extensively for Web or online surveys. However, limited access to internet has been a challenge for online surveys particularly in low income countries such as Ethiopia.

The SAQ to be used for data collection in the current survey is prepared in English in order to make the questions short, simple and understandable for the respondents. We assume that the healthcare workers confidently complete the survey questionnaire in English. The questionnaire has a total of **43** questions with mostly closed ended questions. It asks a series of questions such as general socio-demographics, the types and availability of PPE, use of PPE, and questions related to COVID-19. It will take between 20-30 minutes to complete the questionnaire. The paper questionnaire will be distributed with a cover letter (consent form), introducing the study and explaining the purpose of the survey, instructions on how to complete the questionnaire, and researchers contact information for any questions the respondent might have. There are a total of 07 data collectors assigned for this survey, one data collector per hospital. Assistant data collectors might be assigned to assist the main data collectors at some hospitals depending on the size of the sample sizes allocated to these hospitals. *The overall maximum duration of the data collection is 10 days*.

5. Responsibilities of a supervisor

In this survey, the main role of the supervisor is to organize and coordinate the data collectors and the implementation of the survey. The main roles and responsibilities of the supervisor are listed below:

- Closely work with the research team.
- Coordinate and supervise the overall survey activities.
- Finalize the English questionnaire with the research team.
- Identify and recruit the data collectors.
- Facilitate “Support Letters”, “IRB Approval Letter” and “To Whom It May Concern Letters” for the data collectors.
- Facilitate the contractual agreement for the data collectors.
- Assign data collectors to the institutions/organizations.
- Facilitate transportation for field work in consultation with the College Admin assistant.
- Orient the data collectors about the aims of the study, questionnaire and the data collection procedures including ethical issues by adhering to COVID-19 measures.
- Obtain the final list of wards and the total healthcare workers in each hospital.
- Allocate the sample size to the hospitals and wards in consultation with the research team.
- Closely follow-up and monitor the progress of the data collectors.
- Give solutions to the problems encountered during the field work on the spot or in consultation with the research team.

- Prepare an MS Excel Sheet containing the list of hospitals, list of data collectors (with contact address), number of questionnaires distributed and completed.
- Monitor data collector's log book on the study participants contact number and appointment date and time.
- Summarize the field reports of the data collectors as a final report including the challenges, solutions made and non-response rate on time along with lessons learnt.
- Develop a data entry template with EPI Info/EPI data or other appropriate data management software.
- Recruit an experienced data entry clerk.
- Monitor and supervise the data entry process.
- Clean the data and submit to the research team.

6. Responsibilities of a data collector

In this survey, the main role of the data collector is to distribute the specifically developed questionnaire to potential respondents identified from the above hospitals and later on collect the completed questionnaires. It is not expected to use a probability sampling method to select the participants of this study. However, it is very important to fairly distribute the number of questionnaires to the different wards or units within the selected hospital depending on the size of their healthcare workers. Therefore, the main roles and responsibilities of the data collector include:

- Get orientation about the questionnaire and data collection procedures.
- Read and understand the questionnaire and procedures before departing for data collection.
- Collect the assigned number of questionnaires from the supervisor as per the assigned number of sample size to their designated hospital.
- Sign a contractual agreement at the College of Health Sciences.
- Collect the copy of the "Support Letter" by the University, "IRB Approval letter" and "To Whom May It Concern" letter from the College of Health Sciences.
- Obtain the number of all healthcare workers practicing in the hospital as well as the different wards.
- Identify potential respondents from the selected hospital.
- Introduce your-self to the respondent and provide adequate information about the objectives of the study and data collection procedures.
- Provide adequate information to the respondent about the ethical issues of this study.
- Request the willingness/voluntariness of the respondent to get verbal consent to take part in this study.
- Adequately respond to the questions that might be raised by the respondent.
- Provide the questionnaire to the respondent with adequate information about the nature of the questions and procedures for completing the questionnaire.
- Get contact information about the respondent such as phone number to remind him/her in case the respondent forgets or lost the questionnaire using a logbook.
- Provide clear instructions for returning the completed questionnaire to the data collector or other point of collection.
- Get appointment from the respondent when and where to collect the completed questionnaire using a logbook.

- Collect the completed questionnaires as per the scheduled appointment.
- Check all the returned questionnaires for completeness before leaving the respondent to ask or clarify some questions or problems.
- Develop a strategy for addressing non-response, including how many attempts to reach the respondents face-to-face, by phone and/or other possible methods.
- List the number of wards, the number of questionnaires distributed and the number of completed questionnaires collected using MS Excel Sheet.
- At the end of the data collection, prepare and submit 3-4 pages field report about the data collection situation including challenges encountered and solutions made.
- Submit the completed questionnaires on time along the field report and MS Excel containing the lists of the works done.
- Assist the supervisor as needed.

7. Ethical issues

The following ethical considerations should be strictly followed during the data collection:

- The study protocol for this survey is approved by the IRB of the College of Health Sciences at AAU and the data collection should be conducted as per the protocol.
- All data collectors should have clear understanding of ethical considerations to be followed during this survey.
- Obtain permission to undertake this study from every relevant authority at all levels.
- Data collectors must start with greeting and introducing themselves and why they are there.
- Study participants should be introduced with the objective of the study, benefits, side effects on them or/and their family due to their participation on this study and the time it takes them to complete the questionnaire and ask them for their agreement or consent before distributing the questionnaire to them.
- All data collectors should have the introduction letter and consent form attached to the first page of the questionnaire.
- Verbal consent should be obtained from all participants prior to distributing the questionnaire,
- The agreement and disagreement of the respondents should be respected.
- Assure the respondents that all their answers will be kept confidential and will only be used for the purpose of the survey.
- All data collectors should look professional, courteous and polite, whether in appearance or behavior. Use words such as “please” and “thank you” to motivate the respondents to complete the questionnaire.
- Thank the respondents for their willingness to participate in the study and for their time to complete the questionnaire.
- All data collectors must be aware of COVID-19 infection prevention measures and procedures. These procedures should include proper hand hygiene, correct use of medical or cloth face masks, and maintaining physical distancing to minimize the risk of infection.

THANK YOU!
